# Testing the incremental effectiveness of pay-for-performance to improve implementation of a motivational interviewing brief intervention for substance use disorders in HIV settings: Results of a cluster-randomized type 3 hybrid trial

**DOI:** 10.21203/rs.3.rs-3685134/v1

**Published:** 2023-12-20

**Authors:** Bryan R Garner, Stephen Tueller, Michael Bradshaw, Kathryn Speck, Derek Satre, Carla Rash, Tom Donohoe, Jackie Mungo, Sarah Philbrick, Richa Ruwala, Mathew Roosa, Mark Zehner, James Ford

**Affiliations:** The Ohio State University College of Medicine and Public Health: The Ohio State University College of Medicine; RTI International; RTI International; UNL: University of Nebraska-Lincoln; University of California San Francisco; UConn Health; University of California Los Angeles; RTI International; RTI International; RTI International; Roosa Consulting; University of Wisconsin Madison; University of Wisconsin Madison

**Keywords:** implementation, pay-for-performance, facilitation, motivational interviewing, substance use disorders, HIV, hybrid trials

## Abstract

**Background:**

Substance use disorders (SUDs) have a serious adverse impact on people living with HIV. Previously, using a 39-site dual-randomized type 2 hybrid trial design, findings from the Substance Abuse Treatment to HIV Care Project supported the Implementation and Sustainment Facilitation (ISF) strategy to improve implementation and effectiveness of a motivational interviewing brief intervention (MIBI) for SUD within HIV service settings across the United States (US). Building on this trial, this cluster-randomized type 3 hybrid trial aimed to test the incremental effectiveness of a pay-for-performance (P4P), a form of the “alter incentive/allowance structures” strategy.

**Methods:**

Twenty-six HIV service organizations, their staff participants (N=87), and their client participants (N=341) were cluster-randomized to one of two implementation conditions. The control condition included staff-focused training, feedback, and consultation (TFC) and team-focused implementation and sustainment (ISF). The experimental condition included TFC+ISF as well as P4P (TFC+ISF+P4P). P4P used financial incentives to reward MIBI implementation (US$10 per MIBI delivered) and MIBI implementation at or above a pre-defined level of quality (US$10 per demonstration). In addition to these outcomes, past 4-week changes/reductions in client participant’s days of primary substance use and anxiety symptoms were examined.

**Results:**

The addition of P4P had a large and significant effect on the number of MIBIs implemented (*d*=1.30, p<.05) and reduction in anxiety (*d*=−1.54), but there was no impact on days of substance use. P4P had large effects on MIBI quality (*d*=1.24) and MIBI implementation effectiveness (*d*=1.28), but these were not significant (*p*<.10).

**Conclusions:**

P4P is a form of the “alter incentive/allowance structures” strategy Its function is to reward the implementation of a clinical innovation. Rewarding implementation is consistent with the theory of implementation effectiveness, which suggests implementation climate (i.e., the extent to which implementation is expected, supported, and rewarded) is a key antecedent of implementation effectiveness (i.e., the consistency and quality of implementation). We found that P4P had a significant, positive impact on MIBI implementation in HIV service settings, but client-level outcomes were mixed. Future research should examine the cost-effectiveness of this strategy, as well as to examine the effectiveness of P4P to improve the implementation of other evidence-based innovations.

**Trial registration:**

ClinicalTrials.gov: NCT04687917. Registered 12/18/2020.

## Background

The United States’ Ending the HIV Epidemic initiative, announced in early 2019, put forth an ambitious target of achieving 90% reduction in new HIV infections within 10 years [1]. Substance use disorders (SUDs) continue to pose a barrier to this goal [refs]. For example, in May of 2019, just a few months after the announcement of this initiative and before the COVID-19 pandemic began, Garner and colleagues (2022) [2] conducted a national survey to estimate the prevalence and negative impacts of five different SUDs among people with HIV (PWH) in the United States. Estimates varied by region of the United States, but overall, average prevalence rates were 42.3% for cannabis, 41.9% for alcohol, 34.6% for opioid, 32.2% for methamphetamine, and 28.1% for cocaine. In terms of the negative impacts that each SUD has on HIV care (e.g., being linked to HIV care, being retained in HIV care) and (e.g., having stable housing, being employed), it was estimated the most problematic was methamphetamine and the least problematic was cannabis. When the prevalence rate and negative impact estimates were multiplied to estimate the population-level negative impact, the top three most problematic use disorders were alcohol, methamphetamine, and opioids. Because COVID-19 has been reported to have increased the use of alcohol and other substances as well as the prevalence of anxiety[3–6], the rates of comorbid HIV, SUD and mental health problems may now be even greater.

HIV health and social service settings provide a critical yet under-utilized opportunity to address these adverse impacts [7–10]. According to a review by Hitch and colleagues (2019) [11], SUD screening within many HIV settings is limited. In addition to highlighting the need for HIV settings to conduct systematic SUD screening using validated tools, they noted screening must be accompanied by strategies to facilitate delivery of brief interventions and referral to SUD treatment [11]. This recommendation is consistent with other calls to improve the integration of SUD services within HIV settings [7–10]. More recently, as part of its Overdose Prevention Strategy, the Department of Health and Human Services appropriated $43.7 million dollars of funding in 2022 to support provision of accessible, evidence-based, and culturally appropriate SUD services for people with HIV or at high risk for HIV [12], and further HIV-focused SUD care allocations can be anticipated. The current implementation research, which focused on testing the incremental effectiveness of a pay-for-performance (P4P) strategy to help improve the implementation of an empirically-supported motivational interviewing brief intervention (MIBI) for SUD within HIV settings [13], may help inform how such funding is utilized. The current research may also help inform the field of implementation science, which continues to need effective and cost-effective strategies for improving the implementation of evidence-based innovations within real-world practice settings.

### The Substance Abuse Treatment to HIV Care (SAT2HIV) Project

In 2014, as part of its effort to address comorbid HIV and SUD, the National Institute on Drug Abuse funded the Substance Abuse Treatment to HIV Care (SAT2HIV) Project. Using a dual-randomized type 2 hybrid trial design, 39 HIV service organizations (HSOs) were randomized to one of two implementation conditions [14] and within each HSO, client participants were randomized to one of two intervention conditions [15]. The control implementation condition, based on research by Miller and colleagues (2004) [16], was a staff-focused strategy that provided 2 staff per HSO with training, performance feedback, and on-going consultation in implementing the MIBI with client participants. This is the strategy used by the Addiction Technology Transfer Center (ATTC), an intermediary organization funded by the Center for Substance Abuse Treatment, to prepare staff for implementing motivational interviewing. In addition to this strategy, a team-focused strategy called Implementation and Sustainment Facilitation (ISF) was provided to HSOs and their participating staff (2 MIBI staff and 1–3 leadership staff) randomized to the experimental condition. Grounded in the theory of implementation effectiveness [17–19], which posits implementation climate (i.e., the extent to which implementation is expected, supported, and rewarded) is a key mechanism of change, the ISF strategy focused on optimizing implementation climate during monthly 30–60 minute meetings with the HSO’s MIBI staff and Leadership staff. These meetings were facilitated virtually by an ISF facilitator. Consistent with the Exploration, Preparation, Implementation, and Sustainment (EPIS) framework [20, 21], the focus of ISF meetings varied by EPIS phase. All ISF meetings, however, were guided by the principles of motivational interviewing: engaging, focusing, evoking, and planning [22–24].

The control intervention condition was usual care only (i.e., passive referral [e.g., pamphlet] to a local treatment provider), and the experimental intervention condition consisted of usual care plus a single 15 to 30-minute MIBI. As detailed by Garner and colleagues (2020) (11), compared to the staff-focused training, feedback, and consultation (TFC) control strategy, the addition of the ISF Strategy (TFC + ISF) resulted in significantly greater levels of implementation; both a greater number of MIBIs being implemented and a greater level of MIBI quality (i.e., fidelity) scores. Moreover, compared to usual care only, usual care plus the MIBI significantly reduced daily use of their primary substance for clients within the TFC + ISF condition but not within the TFC only condition [13]. This evidence supporting the effectiveness of the MIBI is consistent with the broader body of evidence supporting the effectiveness of motivational interviewing [25–27].

### Rationale for Testing Pay-For-Performance

P4P is a strategy recommended to improve the quality and value of healthcare and to reduce healthcare disparities [28–30]. P4P is a form of Powell and colleagues’ (2015) [31] “alter incentive/allowance structures” strategy, which they defined as “work to incentivize the adoption and implementation of the clinical innovation.” Multiple meta-analytic studies support the effectiveness of using P4P to improve job performance [32–35]. However, systematic reviews on the use of P4P within healthcare have concluded there remains insufficient rigorous evidence to make any strong conclusions [36–38]. Although limited, there is some rigorous research demonstrating P4P can be an effective and cost-effective strategy for improving the implementation of evidence-based SUD interventions [39–41]. According to a systematic review focused on quality improvement, implementation, and dissemination strategies to improve mental health care for children and adolescents [42], the P4P research conducted by Garner and colleagues (2012) provided the “strongest evidence in the review.” Also supporting the rationale for testing P4P is that it is a strategy that directly addresses the reward dimension of implementation climate – a key mechanism of change according to the theory of implementation effectiveness [17–19].

### Rationale for Trial Design

The current study used a cluster-randomized design (staff clustered within HSO [the unit of randomization]) to minimize the likelihood of contamination across the project’s two implementation conditions, and because the cluster-randomized design may be preferable to alternatives, including the stepped-wedge trial design [43–46]. In addition to the use of a cluster-randomized design, a type 3 hybrid trial was used because of the importance of examining both implementation outcomes and client outcomes [47–51].

### Aims and Hypotheses

**Figure 1:**
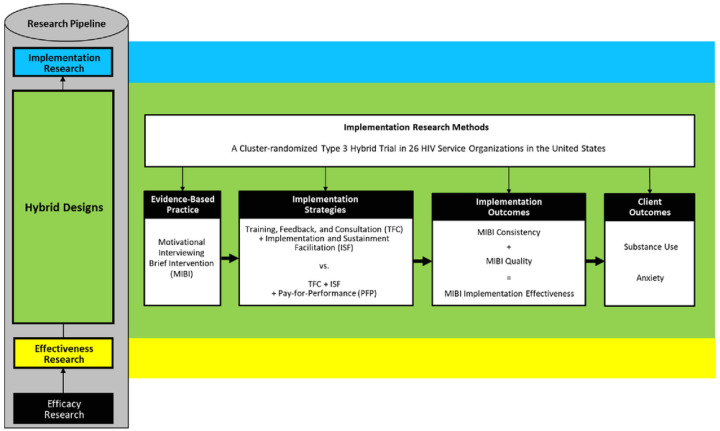
Legend not included with this version.

As illustrated in Fig. 1, the aims of this 26-site cluster-randomized type 3 hybrid trial included testing the effectiveness of P4P for improving implementation outcomes as well as client outcomes. We hypothesized that relative to MIBI staff trained and supported using the TFC + ISF strategy (control condition), which had been the most effective and cost-effective strategy in the prior trial [13, 52], that MIBI staff trained, supported, and rewarded using the TFC + ISF + P4P strategy (experimental condition) would achieve significantly greater: (a) MIBI implementation consistency, (b) MIBI implementation quality, (c) MIBI implementation effectiveness (i.e., the consistency and quality of implementation), and client participants in the experimental condition would achieve significantly greater (d) reductions in days of primary substance use, and (e) reductions in anxiety symptom severity.

## Methods

### Trial Design

The design was a 28-site cluster-randomized, type 3 implementation-effectiveness hybrid trial [48]. Guided by the EPIS framework [20] HSOs (and their staff) were recruited and randomized to one of two implementation strategy conditions (TFC + ISF or TFC + ISF + P4P) during the project’s exploration phase. During the project’s preparation-implementation phase the TFC + ISF strategy was available to HSOs (and their staff) in both conditions. The P4P strategy was only available during the implementation phase and only to MIBI staff cluster-randomized to the TFC + ISF + P4P condition. The HSO was considered to have transitioned from the preparation phase to the implementation phase once a MIBI staff implemented a MIBI with a client participant. The preparation-implementation phase was 12-months for the project’s first cohort and 6-months for the project’s second cohort. This difference was not planned, but was necessitated due to the project being temporarily stopped when the project’s principal investigator changed institutions. Approval and oversight of all research activities was provided by the Institutional Review Board of Advarra.

### Context

The project’s participating HSOs (N = 26) were located in 13 states and the District of Columbia within the United States.

### Participants

#### Staff participants.

For an HSO to be eligible for the project, it had to receive funding from the Health Resources & Services Administrations’ Ryan White program, have two or more staff members (e.g., case manager, behavioral specialist) willing to be trained to implement a MIBI for SUD, and have at least one leadership staff (e.g., director, manager, supervisor) willing to help ensure the MIBI staff were provided sufficient time for participation. The participating HSOs identified individual staff to be invited to participate. Staff were required to be at least 18 years of age. There were not any exclusion criteria for HSO or staff participation. After staff provided informed consent to participate in the project, which was obtained electronically, they completed an online survey that collected background characteristics (e.g., age, biological sex, ethnicity, race, highest level of education). Participants received a US$25 e-gift card upon completion.

#### Client participants.

To be eligible to participate, clients had to be at least 18 years of age and HIV positive. There were no exclusion criteria for client participation. Client participants were recommended to receive a MIBI when they reported unhealthy alcohol use or endorsed two or more of the 11 *Diagnostic and Statistical Manual of Mental Disorders* (DSM-V) criteria [53] for at least one substance during the past 12 months. Client participants received a US$10 e-gift card upon completion of the SUD screener. FMIBI-recommended client participants were asked to complete additional online assessments. These included a pre-MIBI assessment completed within the 24-hour period prior to receiving the MIBI and a follow-up assessment completed 4 weeks after receiving the MIBI. The MIBI was provided at no cost. Participants received e-gift cards upon completion of each assessment (US$20 for pre-MIBI assessment; US$10 for 4-week follow-up assessment). For the 4-week follow-up assessment, participants received a US$10 bonus for completion within 24 hours of the online link being emailed or texted to them or a US$5 bonus for completion within 25–48 hours of the online link being emailed or texted to them.

### Strategies

#### Training, feedback, and consultation.

The TFC strategy, similar to the ATTC strategy in the SAT2HIV Project [13, 14], is a staff-focused strategy composed of three discrete strategies. As part of the current project, MIBI staff were provided access to the project’s MIBI manual, 4-hour online introduction to motivational interviewing training course, and 12-hour virtual training in the MIBI protocol. Although conducted by the same trainer as the SAT2HIV Project, who was and remains part of the Motivational Interviewing Network of Trainers, the 12-hour virtual training was a condensed version of the 2-day in-person training used in the prior trial. This approach was necessitated due to COVID-19 safety protocols. The training content provided as part of the project’s in-person training was condensed into three 4-hour virtual training sessions and was conducted over a four-week period. A one-week break between the second and third training sessions was provided to enable MIBI staff with time to practice the MIBI before the third training session. Free continuing education credits were provided for completion of both the online training and virtual training.

Feedback on the quality of MIBI implementation was provided via a machine-learning based feedback system for motivational interviewing [54]. For each MIBI session at least 10 minutes in length, a MIBI quality score was recorded. The range for each MIBI quality score was 0 to 12, with higher scores indicative of higher quality. MIBI staff were required to demonstrate MIBI proficiency (a score of 4 or higher) via a practice role play session. MIBI role play sessions were conducted with another MIBI staff at their HSO who played the role of the client using a standardized client scenario provided by the project.

In addition to training and feedback, MIBI staff were provided the opportunity to participate in group MIBI consultation meetings. Conducted separately for each condition, these meetings were provided monthly, were 30–60 minutes in length, and were conducted by the trainer who conducted the virtual training sessions. In addition to providing didactic booster training regarding motivational interviewing and our project’s MIBI protocol, these group consultations provided MIBI staff with the opportunity to ask specific questions about how to improve the quality of MIBI implementation with client participants. Each consultation meeting was recorded and a link to the recording was emailed to the MIBI staff in the respective condition.

#### Implementation and sustainment facilitation.

ISF is a team-focused strategy that seeks to optimize MIBI consistency and quality via optimizing the extent to which MIBI staff perceive these dimensions of implementation effectiveness to be expected, supported, and/or rewarded [13, 14]. It is team-focused due to requirement of engagement of staff to deliver the intervention as well as leaders to help address implementation barriers (e.g., competing priorities, insufficient time). To help optimize staff understanding of the implementation initiative and motivation for the training provided as part of the preparation phase, it is recommended that the first ISF strategy meeting be completed prior to the initiation of the staff-focused training strategy. Consistent with other research that has used motivational interviewing as an implementation strategy [55, 56], ISF uses key principles of motivational interviewing (i.e., engaging, focusing, evoking, planning) as a guide. Each monthly ISF strategy meeting was conducted virtually with only one HSO, lasted 30–60 minutes in length, and included three or more of the four motivational interviewing principles. An ISF workbook programmed in Microsoft Excel helped standardize the ISF strategy, document HSO staff attendance, document which motivational interviewing principles were employed, and document any meeting notes and/or action items. The ISF strategy was provided to each HSO by one of four master-level facilitators, two of which had provided ISF as part of the SAT2HIV Project. Weekly ISF team meetings supervised by a PhD-level ISF strategy facilitator were conducted for quality assurance purposes. ISF meetings were recorded and a link to the meeting recording was sent to the HSO’s MIBI staff and leadership staff following each meeting.

#### Pay-for-Performance.

P4P is a staff-focused strategy that utilizes financial incentives and explicitly targets the *reward* dimension of implementation climate – the extent to which implementation is expected, supported, and rewarded [17–19]. For each MIBI session implemented, the MIBI staff received a US$10 incentive. Additionally, for each MIBI session at least 10 minutes in length and that received a 6 + quality rating via a machine-learning based feedback system for motivational interviewing [54], the MIBI staff received a US$10 incentive. This machine-learning based system enabled MIBI staff to receive immediate feedback on session quality, including whether or not their overall score met or exceeded the project’s pre-defined quality target. The project’s target was a score of 6, which was the 80th percentile score obtained by MIBI staff as part of SAT2HIV Project [13].

For the project’s first cohort, P4P incentives that incentivized attendance at the group consultation meetings and ISF meetings were introduced after month 6. Approved by the project’s IRB, MIBI staff were asked to complete a new informed consent form to document their consent to receive a US$30 incentive per monthly group consultation meeting attended and a US$30 incentive per ISF meeting attended. Also approved by the IRB, leadership staff were asked to complete a new informed consent form that documented their consent to receive a US$30 incentive per ISF meeting attended. These additional P4P incentives were only for the HSOs and their participating staff that were randomized to the TFC + ISF + P4P condition. This revised P4P protocol was offered from the start for the project’s second cohort. During the first week of each month, research staff calculated the incentive amount earned by MIBI staff during the prior calendar month, sent a notification email to the staff, and had an e-gift card for the total incentive amount earned sent to the MIBI staff. There was not a limit on the amount MIBI staff were able to earn.

### Intervention

The project’s single session MIBI protocol has been shown to be effective in multiple settings [13, 57]. It was designed to help motivate an individual with comorbid HIV and SUD to change their primary substance use by: (a) examining their reasons for change, (b) receiving feedback about some common negative interactions between substance use and HIV-related physical and mental health issues, (c) increasing the importance or confidence to reduce or stop their primary substance use, and (d) making a plan for change [15].

### Outcomes

#### Implementation outcomes.

Implementation effectiveness is a construct that others have operationally defined as the *consistency*, which is similar to measures like reach [58] and penetration [59], and *quality*, which is similar to measures like fidelity [59] and integrity [60], of innovation implementation by targeted users [17–19]. We defined MIBI consistency as the total number of MIBIs a trained MIBI staff implemented during the project, with no minimum session length. We defined MIBI quality as the total quality score a trained MIBI staff demonstrated during the project. For a MIBI quality score to be generated a MIBI session had to be at least 10 minutes in length. Each MIBI at least 10 minutes in length received a quality score between 0 and 12, with higher scores indicative of higher quality. Following the standardization of each respective measure, these two measures were summed together and standardized to create a measure of implementation effectiveness (i.e., the consistency and quality of implementation) [17–19].

#### Client outcomes.

Consistent with its type 3 hybrid trial design, this project also examined the incremental impact of P4P on client outcomes 4-weeks post-MIBI. The two client outcomes of interest were change in days of primary substance use and change in anxiety symptom severity, both of which were based on client participant’s self-report. They were asked “out of the past 28 days (4 weeks), about how many days did you use [insert their primary substance].” The Generalized Anxiety Disorder 7-item (GAD-7) [61], which ranges from 0 to 21 and where higher scores mean greater severity, was used to assess anxiety during the past two weeks. These client outcomes were aggregated for each MIBI staff. Thus, for each trained MIBI staff we computed two client outcome impact measures. One for their impact on days of primary substance use, and one for their impact on anxiety. A change score was computed for each possible client participant (i.e., those who completed the 4-week follow-up assessment) by subtracting their respective pre-MIBI assessment measure from their respective MIBI follow-up assessment measure. Then, for each respective change score measure we aggregated them to the level of the corresponding MIBI staff. Negative values are preferable as they represent greater reductions in the outcome. If a MIBI staff member was trained but implemented the MIBI with zero client participants, their client impact scores were zero (i.e., no impact, no return on investment).

### Targeted sample size

The project proposed a cluster-randomized trial with 30 HSOs and 90 MIBI staff. Given the project was focused on testing the incremental impact P4P to improve implementation of a MIBI for SUD within HSO there was not a target number of client participants specified, nor was there a minimum number of client participants required per HSO or MIBI staff.

### Randomization sequence generation

For each project cohort, randomization of HSOs (the clusters) was completed by the project’s principal investigator, project coordinator, and statistician. The principal investigator and project coordinator independently matched pairs of HSOs based on information collected as part of an organizational background form. They then met to compare rankings and reach consensus on a final list of match pairs. The final list of matched pairs was given to the statistician who randomized each matched pair to one of the two implementation conditions.

### Blinding

There was no blinding of HSOs or their staff participants to implementation condition. Client participants were blind to implementation condition.

### Statistical methods

Statistical analyses were conducted using an intention-to-implement approach. All MIBI staff who received some component of the TFC strategy or ISF strategy were included in the analyses. All analyses were conducted using SAS version 9.4 [62]. An initial analytic step was to examine the partitioning of variance for each outcome measure. Mixed effects regression analyses were used for the impact of P4P on implementation outcomes and general linear regression analyses were used for the impact of P4P on client outcomes. Doubly robust estimation (i.e., combined propensity score weighting with outcome regression) was used to increase the models robustness to misspecification [63–66]. Factors used to create the staff propensity weight were age, gender, ethnicity, race, education, and experience, both with the organization and in their current position with the organization. All analyses controlled for project cohort. We examined the extent to which there were any significant interactions between condition assignment and the staff factors included in the model. If no significant interaction was found, we focused on the main effect of the implementation condition.

## Results

### Participant flow and recruitment

**Figure 2:**
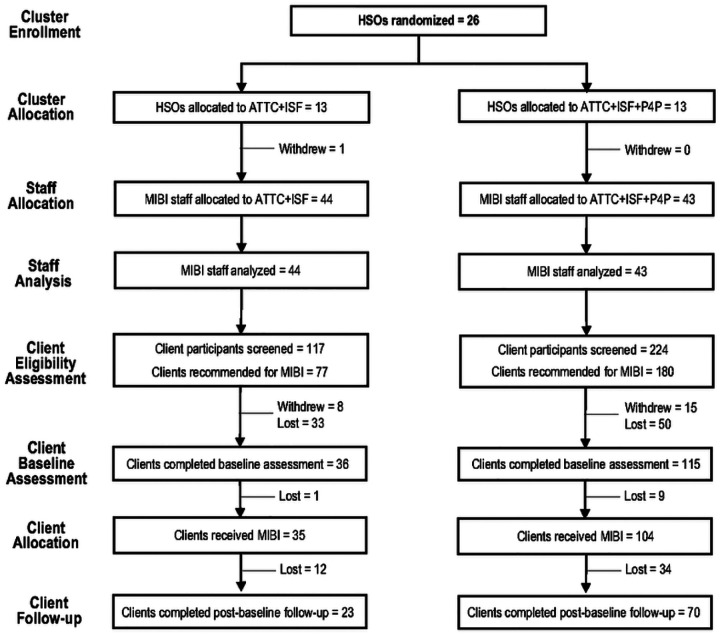
Legend not included with this version.

Two cohorts of HSOs were recruited and randomized as part of the project. The first cohort, which cluster-randomized 12 HSOs, 44 MIBI staff, and 198 client participants, occurred from January 2021 through December 2021. The second cohort, which cluster-randomized 14 HSOs, 43 MIBI staff, and 143 client participants, occurred from November 2021 to April 2022. Figure 2 details the flow of the project’s 26 HSO (87% of targeted sample), 87 MIBI staff (97% of targeted sample), and 341 client participants. As noted previously in the methods section, neither a target number or minimum number of client participants was pre-specified for the HSO or MIBI staff.

Half of the 26 HSOs were randomized to TFC + ISF and the other half were randomized to TFC + ISF + P4P. In the TFC + ISF condition, one HSO withdrew from the project after randomization and before any of the project’s preparation phase strategies (ISF meetings, MIBI training) could be provided. The number of MIBI staff per condition was nearly the same, with 44 in TFC + ISF and 43 in TFC + ISF + P4P. The number of client participants in the TFC + ISF + P4P condition (n = 224) was 91% more than in the TFC + ISF condition (n = 117). The positive screening rate was 14 percentage points higher in TFC + ISF + P4P (80%) than in TFC + ISF (66%). There was little difference between conditions in terms of the number of client participants who withdrew from the study prior to completing the pre-MIBI assessment, with this rate being low for both conditions (10% for TFC + ISF and 8% for TFC + ISF + P4P).

In addition to the SUD screening assessment, client participants were asked to complete a pre-MIBI assessment within the 24-hour period prior to receiving the MIBI. If the pre-MIBI assessment and MIBI were not completed within 30 days of the SUD screen, the MIBI participant was documented as lost. The percentage of client participants lost between completion of the SUD screener and the pre-MIBI assessment was 18 percentage points lower in TFC + ISF + P4P (30%; 50 of 165) than in TFC + ISF (48%; 33 of 69). If using the number of eligible client participants as the denominator, the percentage who received the MIBI was 13 percentage points higher in the TFC + ISF + P4P condition (58%; 104 of 180) than in the TFC + ISF condition (45%; 35 of 77). However, if using the number of client participants who were eligible and completed the pre-MIBI assessment, both conditions had excellent rates: 97% (35 of 36) in the TFC + ISF condition and 90% (104 of 115) in the TFC + ISF + P4P condition. Rates of completion for the 4-week post-MIBI assessment were nearly identical between conditions: 66% (23 of 35) for the TFC + ISF condition and 67% (70 of 104) for the TFC + ISF + P4P condition.

### Participant characteristics

Table 1 presents the baseline characteristics for MIBI staff participants for the overall sample (N = 87) and each condition (43 for TFC + ISF; 44 for TFC + ISF + P4P). Most MIBI staff were female (63%), non-Hispanic (77%), non-White (63%), and did not have a graduate degree (72%). On average, MIBI staff were 40.0 (SD = 12.5) years of age, had 51.4 (SD = 67.6) months of experience with their HSO, and 37.3 months (SD = 53.1) of experience in their current position. Table 2 presents the characteristics of client participants who were recommended for and received the project’s MIBI overall (N = 139). See additional files for the characteristics of all client participants who consented to participate in the study and completed the SUD screener (N = 341) and the subset who were recommended for a MIBI (N = 257).

### Strategy dosage

Table 3 presents information on strategy dosage. In the TFC + ISF strategy condition, the average dose was 12.86 hours (SD = 6.10) of training, 0.68 (SD = 1.61) times receiving feedback, 1.68 times (SD = 1.78) receiving consultation, and 3.52 (SD = 2.00) hours of ISF. Within the TFC + ISF + P4P strategy condition, the average dose was 13.12 hours (SD = 5.74) of training, 2.21 (SD = 1.61) times receiving feedback, 2.72 times (SD = 1.78) receiving consultation, and 3.90 (SD = 2.00) hours of ISF.

### Outcomes

Analyses focused on testing the incremental impact of P4P (TFC + ISF vs. TFC + ISF + P4P) on implementation outcomes (Table 4) and client outcomes (Table 5). None of the staff background characteristics were found to significantly moderate the condition assignment to outcome relationship. This was true for all five outcomes. The addition of P4P had a large effect on all three implementation outcomes but was only statistically significant (*p* < .05) for MIBI consistency, β = 1.30, 95% confidence interval (CI) = [0.002, 2.6], *p* = .0497). For MIBI quality and MIBI implementation effectiveness, the results were β = 1.24, 95% CI = [−0.18, 2.7], (*p* = .08) and β = 1.28, 95% CI = [−0.08, 2.6], (*p* = .06), respectively. In terms of client outcomes, overall reduction in days of primary substance was greater for MIBI clients assigned to the TFC + ISF + P4P condition, β = −0.34, 95% CI = [−1.63, 0.95], but was not statistically significant (*p* = .59). The addition of P4P had a significant impact on reduction in client anxiety symptoms, β = −1.54, 95% CI = [−2.96, −0.11], *p* = .04). However, there was a significantly greater overall reduction in level of client anxiety for those whose MIBI staff who were female (β = −1.66, 95% CI = [−3.12, −0.21], *p* = .03) and/or Hispanic (β = −2.63, 95% CI = [−4.47, −0.79], *p* = .007).

## Discussion

There is growing interest in improving the integration of SUD services within HIV service settings [11, 9, 67–69]. Consistent with a systematic review highlighting the need for studies that “move beyond discrete training events and towards longer term coaching-type activities focused on implementation” [70] Garner and colleagues (2020) [13] found adding the ISF strategy (a team-focused coaching-type strategy) on top of the TFC strategy (a staff-focused training-type strategy) significantly improved both (a) the implementation of a MIBI for SUD within HSOs, and (b) the effectiveness of the MIBI in reducing substance use. Building on this research, the aim of the current 26-site cluster-randomized type 3 hybrid trial was to test the incremental effectiveness of P4P (TFC + ISF vs. TFC + ISF + P4P) on both implementation outcomes and client outcomes.

In the current study, addition of P4P had a large and statistically significant impact on MIBI consistency (the number of MIBIs implemented), with the TFC + ISF + P4P condition implementing more than triple the number implemented within the TFC + ISF condition: 115 vs. 36. This large effect of P4P on implementation is consistent with prior research that found P4P to improve implementation of an adolescent substance use intervention [40]. Although not an aim of the current project, future research examining the incremental cost-effectiveness of this P4P strategy may be warranted given how cost-effective the P4P was found to be in the trial with adolescents; a 5% increase in cost led to a 325% increase in clients receiving the pre-defined dosage of treatment [41].

The addition of the P4P strategy also had a large effect on the overall level of MIBI quality achieved by MIBI staff, but this effect was not statistically significant according to our pre-specific criteria of *p* < .05. Rather, in contrast to the statistically significant effect found for MIBI consistency, the impact of P4P on MIBI quality was slightly smaller and short of achieving statistical significance. This was also the case for MIBI implementation. Our finding that P4P had a slightly less positive effect on quality than on quantity is in line with findings from a recent meta-analysis by Kim and colleagues (2022) [35]. More specifically, the meta-analysis by Kim et al. found that “the effect of incentives on performance was consistently and meaningfully larger for performance quantity than for quality,” which is a finding they highlighted as being consistent with cognitive evaluation theory [71–73] and its later incorporation into self-determination theory [74–77].

Regarding impact on client outcomes, MIBI staff in the TFC + ISF + P4P condition had slightly greater overall changes/reductions in days of primary substance use by clients compared to the TFC + ISF condition (*d* = − .34), but this was not at all close to being statistically significant (*p* = .59). Although also not statistically significance (*p* = .11), we did find MIBI staff who had more experience with their organization had greater overall changes/reductions in days of primary substance use by clients. However, given that more experience working in the HSO was associated with poorer MIBI quality (though not statistically significant), we believe it may be possible that MIBI staff with greater experience working in their HSO were able to establish stronger therapeutic rapport/alliance with their clients; a relationship that much research has found to be one of the best predictors of improved client outcomes. Although beyond the scope of the current research, it may therefore be warranted for future research to rate MIBI sessions for therapeutic rapport/alliance and to examine its relationship with client changes/reductions in days of substance use.

Despite not being the focus of the MIBI sessions, we did find that client participants in the TFC + ISF + P4P condition had significantly greater changes/reductions in anxiety symptom severity (*d* = −1.54; *p* = .04) compared to those in the TFC + ISF condition. Although this finding is encouraging, it must be acknowledged that the more significant predictors of this outcome was if the MIBI staff was female (*p* = .03) or Hispanic (*p* = .007). There is research that has previously suggested there being a “female effect” [78], but we are not aware of research that has found similar effect for interventionists who identify as Hispanic. Future research exploring this issue may therefore be warranted.

### limitations

The current research was not without limitations. First, while both implementation conditions were affected, conducting the research during COVID-19 was not ideal and may have limited the extent to which the MIBI was able to be implemented. Indeed, although conducting the MIBI via phone or virtually was also an option as part of the earlier trial [13], more MIBIs had to be conducted this way due to COVID-19 and may have limited the quality of MIBI implementation. Second, client outcomes were limited to two measures that were based on self-report. Third, only about two-thirds of follow-up assessments were completed, which was lower than the 70% rate we had targeted as a minimum follow-up rate, and therefore does adversely impact generalizability of client-level findings.

## Conclusions and recommendations

The consistency and quality of implementation (i.e., implementation effectiveness) is believed to be the greatest when it is expected, supported, and rewarded [17–19]. Building upon our prior research [13, 40, 41, 52], the current study findings support the effectiveness of using P4P to improve implementation of a MIBI for SUD within HIV service settings. An examination of cost-effectiveness is needed, as is an examination of how effective and cost-effective P4P may be for improving the implementation of other clinical innovations that are both evidence-based and underutilized in real-world practice settings.

## Figures and Tables

**Table 1:** MIBI Staff demographic characteristics

	Overall (N = 87)		TFC + ISF (N = 44)		TFC + ISF + P4P (N = 43)	
	N	%	N	%	N	%
Age						
18–24	4	4.6	2	4.6	2	4.7
25–34	35	40.2	23	52.3	12	27.9
35–44	17	19.5	7	15.9	10	23.3
45–54	13	14.9	5	11.4	8	18.6
55–64	15	17.2	7	15.9	8	18.6
65 and older	3	3.5	0	0.0	3	7.0
Female	58	66.7	31	70.4	27	62.8
Hispanic	27	31.0	17	38.6	10	23.3
White	39	44.8	23	52.3	16	37.2
Graduate degree or higher	34	39.1	22	50.0	12	27.9
Experience at current organization						
12 months or less	29	33.3	15	34.1	14	32.6
13–24 months	6	6.9	2	4.6	4	9.3
25–60 months	32	36.8	19	43.2	13	30.2
61–120 months	9	10.3	6	13.6	3	7.0
121 + months	11	12.6	2	4.6	9	20.9
Tenure at current position						
12 months or less	32	37.2	16	37.2	16	37.2
13–24 months	13	15.1	6	14.0	7	16.3
25–60 months	31	36.1	19	44.2	12	27.9
61–120 months	4	4.7	2	4.7	2	4.7
121 + months	6	7.0	0	0.0	6	14.0
Baseline measures						
Implementation Climate scale score (mean (SD))	87	2.2 (0.9)	44	2.1 (0.9)	43	2.4 (0.8)
MI knowledge and experience (mean (SD))	87	5.4 (16)	44	5.0 (13)	43	5.9 (1.8)
Setting Intervention Fit Scale Score (mean (SD))	87	17.3 (4.0)	44	16.5 (4.1)	43	18.2 (3.9)
Organizational Readiness for Change (mean (SD))	87	4.4 (0.6)	44	4.4 (0.6)	43	4.5 (0.6)

**Note:** TFC = Training, Feedback, and Consultation; ISF = Implementation & Sustainment Facilitation; MIBI = motivational interviewing-based brief intervention, P4P = Pay For Performance

**Table 2:** Client demographic characteristics (Received MIBI and Completed Follow-up Interview)

	Overall (N = 93)		TFC + ISF (N = 23)		TFC + ISF + P4P (N = 70)	
	N	%	N	%	N	%
**Age**						
18–24	0	0.0	0	0.0	0	0.0
25–34	11	11.8	3	13.0	8	11.4
35–44	21	22.6	7	30.4	14	20.0
45–54	26	28.0	6	26.1	20	28.6
55–64	29	31.2	6	26.1	23	32.9
65 and older	6	6.5	1	4.4	5	7.1
**Female**	27	29.0	4	17.4	23	32.9
**Hispanic**	30	32.3	9	39.2	21	30.0
**White**	38	40.9	10	43.5	28	40.0
**Change in Outcomes From Baseline to Follow-up**						
Days of Change in Primary Substance Use (mean (SD))	92	−3.32 (8.0)	22	−2.2 (7.4)	70	−3.7 (8.2)
Change in Anxiety GAD7 scale score (mean (SD))	92	−1.16 (5.1)	92	−0.26 (5.2)	92	−1.5 (5.0)

**Note:** TFC = Training, Feedback, and Consultation; ISF = Implementation & Sustainment Facilitation; MIBI = motivational interviewing-based brief intervention, P4P = Pay For Performance

**Table 3:** Strategy dosage

	Overall (N = 44)		Control (N = 21)		Experimental (N = 23)	
Cohort 1	Mean	SD	Mean	SD	Mean	SD
Training (hours)	13.27	5.89	14.10	4.88	12.52	6.60
Feedback (times)	1.68	3.08	0.71	2.00	2.57	3.59
Consultation (minutes)	136.36	159.96	100.00	111.61	169.57	187.79
ISF (minutes)	246.49	23.19	225.76	137.41	267.22	170.20
P4P earned (dollars)	N/A	N/A	N/A	N/A	26.52	37.60

	Overall (N = 43)		Control (N = 23)		Experimental (N = 20)	
Cohort 2	Mean	SD	Mean	SD	Mean	SD
Training (hours)	12.70	5.94	11.74	6.83	13.80	4.47
Feedback (times)	1.19	2.46	0.65	1.13	1.80	3.30
Consultation (minutes)	126.98	133.36	101.74	102.31	156.00	156.92
ISF (minutes)	196.45	14.71	197.70	103.78	195.20	124.59
P4P earned (dollars)	N/A	N/A	N/A	N/A	31.50	58.16

	Overall (N = 87)		Control (N = 44)		Experimental (N = 43)	
Overall	Mean	SD	Mean	SD	Mean	SD
Training (hours)	12.99	5.93	12.86	6.10	13.12	5.74
Feedback (times)	1.44	2.80	0.68	1.61	2.21	3.47
Consultation (minutes)	131.72	147.49	100.91	106.85	163.26	174.25
ISF (minutes)	222.28	23.31	211.09	120.39	233.72	153.35
P4P earned (dollars)	N/A	N/A	N/A	N/A	28.84	48.33

**Note:** ISF = Implementation & Sustainment Facilitation; P4P = pay for performance

**Table 4:** Results of analyses of the impact of the P4P strategy on implementation outcomes

	Full Sample		
	Estimate (95% CI)	SE	p value
**MIBI Count**			
ATTC + ISF + P4P	1.30 (0.002,2.6)	0.56	0.0497**
Cohort 1	0.57 (−0.67,1.80)	0.54	0.32
Age	−0.002 (−0.04,0.04)	0.02	0.91
Female	−0.44 (−1.18,0.31)	0.32	0.22
Hispanic	0.37 (−1.03,1.76)	0.60	0.56
White	0.52 (−0.44, 1.47)	0.41	0.25
Graduate degree or higher	0.32 (−0.81,1.45)	0.49	0.53
Experience at current position	0.004 (−0.009,0.02)	0.005	0.52
Experience at current organization	−0.004 (−0.02,0.006)	0.005	0.37
**MI Proficiency**			
ATTC + ISF+ P4P	1.24 (−0.18,2.67)	0.62	0.08*
Cohort 1	0.36 (−1.00,1.71)	0.59	0.56
Age	−0.006 (−0.05,0.04)	0.02	0.75
Female	−0.54 (−1.27,0.20)	0.32	0.13
Hispanic	0.39 (−1.08,1.85)	0.64	0.18
White	0.60 (−0.35,1.56)	0.41	0.24
Graduate degree or higher	0.63 (−0.52,1.78)	0.50	0.50
Experience at current position	0.004 (−0.009, 0.02)	0.005	0.23
Experience at current organization	−0.006 (−0.02,0.005)	0.59	0.56
**Implementation Effectiveness**			
ATTC + ISF+ P4P	1.28 (−0.08,2.64)	0.59	0.06*
Cohort 1	0.46 (−0.83,1.76)	0.56	0.43
Age	−0.004 (−0.05,0.04)	0.02	0.83
Female	−0.49(−1.23,0.25)	0.32	0.17
Hispanic	0.38 (−1.05,1.81)	0.62	0.55
White	0.56 (−0.39,1.51)	0.41	0.21
Graduate degree or higher	0.48 (−0.66,1.62)	0.49	0.36
Experience at current position	0.004 (−0.008,0.02)	0.005	0.50
**MIBI Count**			
Experience at current organization	−0.005 (−0.02,0.005)	0.004	0.29

**Note:** CI = Confidence Interval; MIBI = motivational interviewing-based brief intervention; ATTC = Addiction Technology Transfer Center; ISF = Implementation & Sustainment Facilitation, P4P = Pay For Performance.

* p <.0.10,

** p<0.05,

*** p<0.01

**Table 5:** The incremental impact of the P4P strategy on change in client-level outcomes

	Full Sample		
	Estimate (95% CI)	SE	p value
**Sum of PSU Days Changed – All MIBI Staff**			
ATTC + ISF+ P4P	−0.34 (−1.63,0.95)	0.62	0.59
Cohort 1	−0.47 (−1.74,0.79)	0.61	0.45
Age	0.007 (−0.06,0.07)	0.03	0.82
Female	0.34 (−0.98,1.66)	0.64	0.60
Hispanic	1.00 (−0.66,2.67)	0.80	0.22
White	0.16 (−1.32,1.65)	0.72	0.82
Graduate degree or higher	0.93 (−0.57,2.43)	0.72	0.21
Experience at current position	0.01 (−0.008,0.03)	0.009	0.26
Experience at current organization	−0.01 (−0.03,0.003)	0.007	0.11
**Sum of GAD7 Score Changed – All MIBI Staff**			
ATTC + ISF+ P4P	−1.54 (−2.96,−0.11)	0.69	0.04**
Cohort 1	−0.36 (−1.76, 1.03)	0.67	0.60
Age	−0.009 (−0.08, 0.06)	0.04	0.80
Female	−1.66 (−3.12, −0.21)	0.70	0.03**
Hispanic	−2.63 (−4.47,−0.79)	0.89	0.007***
White	−0.50 (−2.13,1.14)	0.79	0.54
Graduate degree or higher	−0.52 (−2.18, 1.13)	0.80	0.52
Experience at current position	−0.008 (−0.03, 0.01)	0.01	0.41
Experience at current organization	0.008 (−0.009, 0.03)	0.008	0.31

**Note:** CI = Confidence Interval; MIBI = motivational interviewing-based brief intervention; ATTC = Addiction Technology Transfer Center; ISF = Implementation & Sustainment Facilitation, P4P = Pay For Performance.

* p <.0.10,

** p<0.05,

*** p<0.01

## Data Availability

Upon reasonable request, which should be made to the corresponding author, study data or materials may be made available.

## Abbreviations

ATTC: Addiction Technology Transfer Center
CI: Confidence Interval
DSM-V: Diagnostic and Statistical Manual of Mental Disorders
EPIS: Exploration, Preparation, Implementation, Sustainment
GAD-7: Generalized Anxiety Disorder 7-item
HIV: Human Immunodeficiency Virus
HSO: HIV service organization
ISF: Implementation and Sustainment Facilitation
MIBI: Motivational Interviewing Brief Intervention
P4P: Pay for Performance
PWH: People with HIV
SAT2HIV: Substance Abuse Treatment to HIV Care
SD: Standard Deviation
SUD: Substance Use Disorder
TFC: Training, Feedback, and Consultation
US: United States
